# What is a manual of procedures and why do we need one?

**DOI:** 10.36416/1806-3756/e20240137

**Published:** 2024-05-08

**Authors:** Juan C Calderon, Juliana C Ferreira, Cecilia M Patino

**Affiliations:** 1. Methods in Epidemiologic, Clinical, and Operations Research-MECOR-program, American Thoracic Society/Asociación Latinoamericana del Tórax, Montevideo, Uruguay.; 2. Universidad Espíritu Santo, Samborondón, Ecuador.; 3. Respiralab Research Group, Guayaquil, Ecuador.; 4. Divisão de Pneumologia, Instituto do Coração, Hospital das Clínicas, Faculdade de Medicina, Universidade de São Paulo, São Paulo (SP) Brasil.; 5. Department of Preventive Medicine, Keck School of Medicine, University of Southern California, Los Angeles (CA) USA.

## PRACTICAL SCENARIO

At the beginning of this millennium, the PLATINO multi-country cross-sectional study was planned by a group of researchers to measure the burden of COPD among adults from major cities in Latin America (São Paulo, Mexico City, Montevideo, Caracas, and Santiago).^1^ The investigators measured the prevalence of COPD and its risk factors using a standardized paper questionnaire and standardized pulmonary function methodology. The authors reported that the prevalence of COPD was higher than expected and ranged from 7.8% (95% CI, 5.9-9.7) in Mexico City to 19.7% (95% CI, 17.2-22.2) in Montevideo, Uruguay.^2^ To yield accurate results, investigators collected data across study sites using the same methodological standards by writing and implementing a manual of procedures (MOP).^1^

## WHAT IS A MOP?

Although the research process begins with an idea derived from an observation, the next steps include writing a research question, a protocol, and a detailed manual of procedures to guarantee accurate data collection process, as well as a comprehensive data analysis plan to provide reliable answers to important research questions that impact population health. At each step of the research protocol, inconsistencies in data collection procedures can lead to excess variation and/or error, jeopardizing the validity and reliability of the results. Therefore, the MOP is necessary to document and standardize all research procedures. The research process evolves from the initial research protocol into a fully detailed operations manual, describing every procedure stated in the protocol, and encompasses study organization, policies, participant recruitment and enrollment, randomization, blinding procedures, variable measurements, quality control, data management practices, and the statistical plan (Table 1).

**Table 1:** Outline of a manual of procedures (MOP).

Procedure	Example
Introduction	Overview of study goals and rationale
Study protocol	In many cases, the study protocol is the first document included in the MOP
Organization	Description of the team of investigators and research study units in multicentric studies
Recruitment and enrollment procedures	Procedures to identify and recruit potentially eligible patients and check for inclusion and exclusion criteria; informed consent
Randomization & blinding	Procedure used to randomize patients using a website
Ethical considerations	Protection of participant confidentiality and privacy Compliance with ethical guidelines and regulations
Clinic visits	Which variables are to be measured at baseline and at specific time-points during follow-up and how they will be measured
Intervention/exposure/predictors & comparison group	Detailed description of the intervention and the control groups (for unblinded studies)
Study variables	Detailed instructions on how the primary outcome variable and other important variables will be measured, including adverse events that will be measured
Quality control	Responsibilities, training of data collection team, and equipment calibration and maintenance
Data management	How data will be collected and stored; confidentiality; plan for backups
Data analysis	A detailed data analysis plan
Emergency procedures	Protocols for managing medical emergencies during study visits
Communication plan	Procedures for communication among study staff, investigators, and participants Contact information for key study personnel
Appendices	Questionnaires, forms

All types of research study designs need a MOP, but MOPs are especially relevant for randomized clinical trials and cohort studies, because the types of procedures used in these studies are complex and need to be well standardized, but descriptive studies are not exceptions.^1^ In the case of the PLATINO study, despite cultural, social, and economic disparities among Latin-American cities, the use of a MOP, which included standardized instructions on how to perform a spirometry test, enabled the execution of high-quality research, addressing crucial research questions and establishing the prevalence and severity of COPD in major cities of Latin America.

## WHAT GOES IN A MOP?

All study procedures should be included in the MOP. In the PLATINO study, for example, there was a section dedicated to training and certification of study staff to perform spirometry. In addition, there was detailed description on how to perform an acceptable spirometry test and the accepted variability for selected maneuvers. Even simpler procedures, such as the technique to measure anthropometric variables were detailed, as well as the sampling procedure. As individuals were recruited from Portuguese and Spanish speaking cities, the questionnaires were validated for both languages. The MOP meticulously describes procedures, anticipates data variability, addresses recruitment and follow-up errors, and delineates strategies to minimize bias and maximize quality control, assuring validity and generalizability to the target population. 

## TIPS TO WRITE A MOP

- Describe in depth all the procedures used in the study, like a recipe.
- Be systematic and anticipate all possible misunderstandings; be accurate.
- Ask for feedback from the study team to check for any errors.
- Implement strategies to minimize errors and plan for possible solutions.
- Remember, all investigators and study staff will consult the MOP and are expected to follow the instructions.
